# Consequences of untreated mental health in Bangladesh during COVID-19 pandemic

**DOI:** 10.1186/s42269-022-00731-1

**Published:** 2022-02-23

**Authors:** Saad Ahmed Sami

**Affiliations:** Department of Pharmacy, Faculty of Biological Sciences, University of Chittagong, Chittagong, 4331 Bangladesh

To the Editor,

The terms ‘lockdown’, ‘quarantine’ and ‘isolation’ were alien to most people of Bangladesh before the emergence of COVID-19 infection. Geographically, being closed to disease epicenter China, Bangladesh also fall victim to this deadly virus and still undergoing critical conditions. This catastrophic situation has negatively impacted physical and mental health; disrupted economy, education, social aspects.

Mental health is an important factor for the personal or professional development of an individual. People with mental health disorders are more susceptible to other diseases and decrease the work capacity of a person. The enforcement of lockdown created immense mental pressure among people. It resulted in many people losing their job, livelihood and becoming bankrupt. COVID-19 related containment measures such as social-distancing, quarantine, isolation increased the risk of serious psychiatric problems. The reduced social interactions and increased loneliness exacerbated depression, anxiety, panic, fear, suicidal-tendency and traumatic stress. Refusal to sit next to someone on public transport with suspected cold, entry refusal into the house and anxiety-driven panic buying were observed among people (Wang et al. 2020; Islam et al. 2020).

Xenophobia and the agony of abject poverty have led many indigent individuals to commit suicide during COVID-19. For instance, a man returned to his village from Dhaka in the early lockdown period. Avoidance and negative-attitudes of villagers due to his fever-cold and weight loss compelled him to commit suicide but autopsy report found no COVID-19 in the victim. Another person committed suicide because of unemployment and the pressure of unpaid debts. It was reported that most people were stressed by thinking about the family member and relatives, future plans and financial difficulties (Bhuiyan et al. 2020; Mamun and Griffiths 2020). The normal psychological development of children got hampered due to this pandemic-induced loss of companionship and separation from infected parents. Children spending extra-time on electric devices have succumbed to mental health problems. An online survey on children after one month of quarantine in May, 2020 reported subthreshold (43%), mild (30.5%), moderate (19.3%), and severe (7.2%) mental health disturbances (depression, anxiety, and sleeping disorder) among participants. If one considers the urban lifestyle a solution, then you will be surprised to know that mass individuals were from urban areas (above 60%) in these cases. At that state, the question was “Life vs Livelihood”. Lockdowns that seemed draconian when instigated for the first time became very common and flawed over time. People were working outside in large numbers and were not afraid to get infected by COVID-19 (Islam et al. 2020; Yeasmin et al. 2020).

Under strict infection measures, mental health professionals were not allowed to work alongside physicians in most hospitals in Bangladesh. Therefore, the psychological interventions controlled by doctors and nurses resulted in reduced effectiveness of the treatment. Some scientifically approved measures could be taken in these circumstances like multidisciplinary mental health team formation involving professionals, clear communication and regular updates on disease-state with social-psychological counseling services (Xiang et al. 2020). Mental health is as important as physical health as it can lead to many critical disorders beyond this pandemic. Compassion and solidarity may work as a medicine to tackle COVID-19 mental health challenges and maintain good mental health.

## Data Availability

Not applicable.
